# High‐Power Performance of Textured Piezoelectric Ceramics Through Synergistic A‐Site Donor and B‐Site Acceptor Doping

**DOI:** 10.1002/smtd.202502326

**Published:** 2026-04-01

**Authors:** Minwoo Kim, Dong‐Gyu Lee, Il‐Ryeol Yoo, Byeong‐Jae Min, Ye Rok Choi, Hyun Soo Kim, Sunghoon Hur, Heemin Kang, Sahn Nahm, Jungho Ryu, Yongke Yan, Jeong Min Baik, Kyung‐Hoon Cho, Hyun‐Cheol Song

**Affiliations:** ^1^ Department of Materials Science and Engineering Korea University Seoul Republic of Korea; ^2^ School of Materials Science and Engineering Kumoh National Institute of Technology Gumi Republic of Korea; ^3^ Electronic and Hybrid Materials Research Center Korea Institute of Science and Technology (KIST) Seoul Republic of Korea; ^4^ Department of Bioengineering University of California Los Angeles CA USA; ^5^ KIST‐SKKU Carbon‐Neutral Research Center Sungkyunkwan University (SKKU) Suwon Republic of Korea; ^6^ School of Advanced Materials Science and Engineering Sungkyunkwan University (SKKU) Suwon Republic of Korea; ^7^ School of Materials Science and Engineering Yeungnam University Gyeongsan Republic of Korea; ^8^ Electronic Materials Research Laboratory Key Laboratory of the Ministry of Education and International Center for Dielectric Research School of Electronic Science and Engineering Xi'an Jiaotong University Xi'an China

**Keywords:** combinatorial doping, morphotropic phase boundary (MPB), PIN‐PMN‐PT piezoceramics, templated grain growth (TGG), ultrahigh figure‐of‐merit (d_33_×Q_m_)

## Abstract

Temperature stability is a critical factor in high‐power applications due to the substantial heat generated during high‐frequency resonant operation. While properties such as the electromechanical quality factor (*Q*_m_) and Curie temperature (*T*_C_) are essential for ensuring thermal robustness, enhancing the piezoelectric constant (*d*_33_) is equally important for improving overall performance. However, these properties often exhibit trade‐offs, making simultaneous enhancement a significant challenge. In this study, a complementary doping strategy was adopted by introducing Eu ion as an A‐site donor and Mn ion as a B‐site acceptor into the PIN–PMN–PT system. This approach effectively mitigated cation–oxygen vacancy recombination through spatial separation. Furthermore, the templated grain growth (TGG) method was employed to align domains, thereby further enhancing the piezoelectric properties. As a result, 0.24PIN–0.46PMN–0.30PT textured ceramics co‐doped with 2 mol% Mn and 1.5 mol% Eu ions exhibited outstanding performance metrics: *d*_33_ of 508 pC/N, a planar coupling coefficient (*k*_p_) of 64%, and *Q*_m_ exceeding 850, while maintaining *T*_C_ above 170°C. The resulting transducer Figure‐of‐merit (*d*_33_ × *Q*_m_) surpasses 400 000, establishing a new benchmark for high‐power piezoelectric materials and demonstrating strong potential for next‐generation transducer technologies.

## Graphical abstract

A/B‐site co‐doping combined with templated grain growth enables synergistic defect and domain engineering in textured PIN–PMN–PT ceramics. The approach simultaneously enhances piezoelectric response and mechanical quality factor, achieving a balanced soft–hard characteristic. The resulting materials exhibit a high transducer figure‐of‐merit and improved thermal stability, offering a promising strategy for high‐power piezoelectric applications.

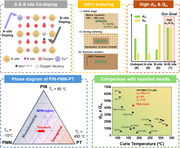

## 1 Introduction

The continuous expansion of energy harvesting technologies and high‐precision electromechanical systems has intensified the demand for piezoelectric materials capable of operating reliably under increasingly demanding conditions. Perovskite ABO_3_ lead‐based piezoelectric ceramics have emerged as critical components in high‐power energy conversion applications, powering diverse technologies from medical ultrasound transducers to industrial ultrasonic motors and transformers [1, 2, 3, 4]. Despite their widespread adoption, these materials face fundamental challenges when operated under high electric fields near resonance frequencies, where significant heat generation occurs—leading to reduced efficiency, performance degradation, and potential system failure. This thermal limitation represents a critical bottleneck in advancing high‐power piezoelectric technologies toward their theoretical performance limits.

The development of next‐generation high‐power piezoelectric materials requires simultaneous optimization of multiple competing properties: elevated piezoelectric strain (or charge) coefficient (*d*_33_) for enhanced displacement, high mechanical quality factor (*Q*_m_) to minimize heat generation, and sufficient Curie temperature (*T*_C_) to maintain operational stability across varying thermal conditions [5, 6, 7]. The mechanical quality factor deserves particular attention as it directly correlates with reduced heat generation and enhanced displacement amplification at resonance—critical factors for high‐power applications [8]. Furthermore, efficient energy conversion demands both elevated piezoelectric constat (*d*_33_) and electromechanical coupling factors (*k*_p_) to ensure maximum energy transfer and vibration velocity during operation [9, 10, 11, 12, 13, 14, 15].

The fundamental challenge in piezoelectric materials engineering stems from the inherent inverse relationship between “soft” characteristics (high *d*_33_) and “hard” characteristics (high *Q*_m_). This inverse relationship has long represented a materials science paradox, forcing engineers to compromise between displacement capability and thermal stability. Conventional approaches have segregated piezoelectric ceramics into distinct categories: “soft” materials optimized for sensing applications where sensitivity dominates, and “hard” materials designed for high power applications where thermal stability prevails. Breaking this paradigm requires innovative approaches to defect chemistry and microstructural engineering.

Recent investigations into doping mechanisms have revealed promising pathways toward reconciling these competing properties. Rare earth elements—particularly Sm^3+^ ions—introduced as donor dopants at the A‐site demonstrate remarkable ability to enhance *d*_33_ values by promoting domain wall motion and increasing local structural heterogeneity within the perovskite lattice [16, 17, 18]. Concurrently, conventional acceptor doping with MnO_2_ at the B‐site introduces the hardening characteristics necessary for high‐power stability [9, 19, 20, 21, 22]. Rather than allowing these dopants to counteract each other's effects, we propose that strategic spatial separation of doping sites within the crystal structure can preserve the beneficial aspects of both doping mechanisms simultaneously [2, 5, 23].

This spatial separation approach does not directly prevent donor‐acceptor recombination but rather minimizes the extent to which such recombination suppresses the formation of functional defects—specifically oxygen vacancies and cation vacancies that govern domain wall dynamics. By maintaining controlled concentrations of these critical defects, we hypothesize that materials can simultaneously exhibit enhanced piezoelectric response and thermal stability, effectively circumventing the traditional *d*_33_‐*Q*_m_ trade‐off that has constrained piezoelectric performance [24]. Similar approaches combining defect engineering and crystallographic texturing have been actively explored in recent high‐power piezoelectric studies, demonstrating improved mechanical quality factors and thermal stability [25, 26, 27].

Complementing this chemical approach, microstructural engineering through grain texturing offers an additional dimension for property enhancement. By facilitating domain alignment through controlled grain orientation, texturing can further amplify piezoelectric response while preserving the hardening characteristics established through chemical modification. The ternary ferroelectric system Pb(In_1/2_Nb1_1/2_)O_3_‐Pb(Mg_1/3_Nb_2/3_)O_3_‐PbTiO_3_ (PIN‐PMN‐PT) presents an ideal platform for implementing these combined strategies due to its intrinsically high Curie temperature exceeding 200°C and compositional flexibility [28]. More importantly, this system demonstrates exceptional compatibility with the Templated Grain Growth (TGG) process, enabling precise microstructural control for domain engineering [29].

**FIGURE 1:**
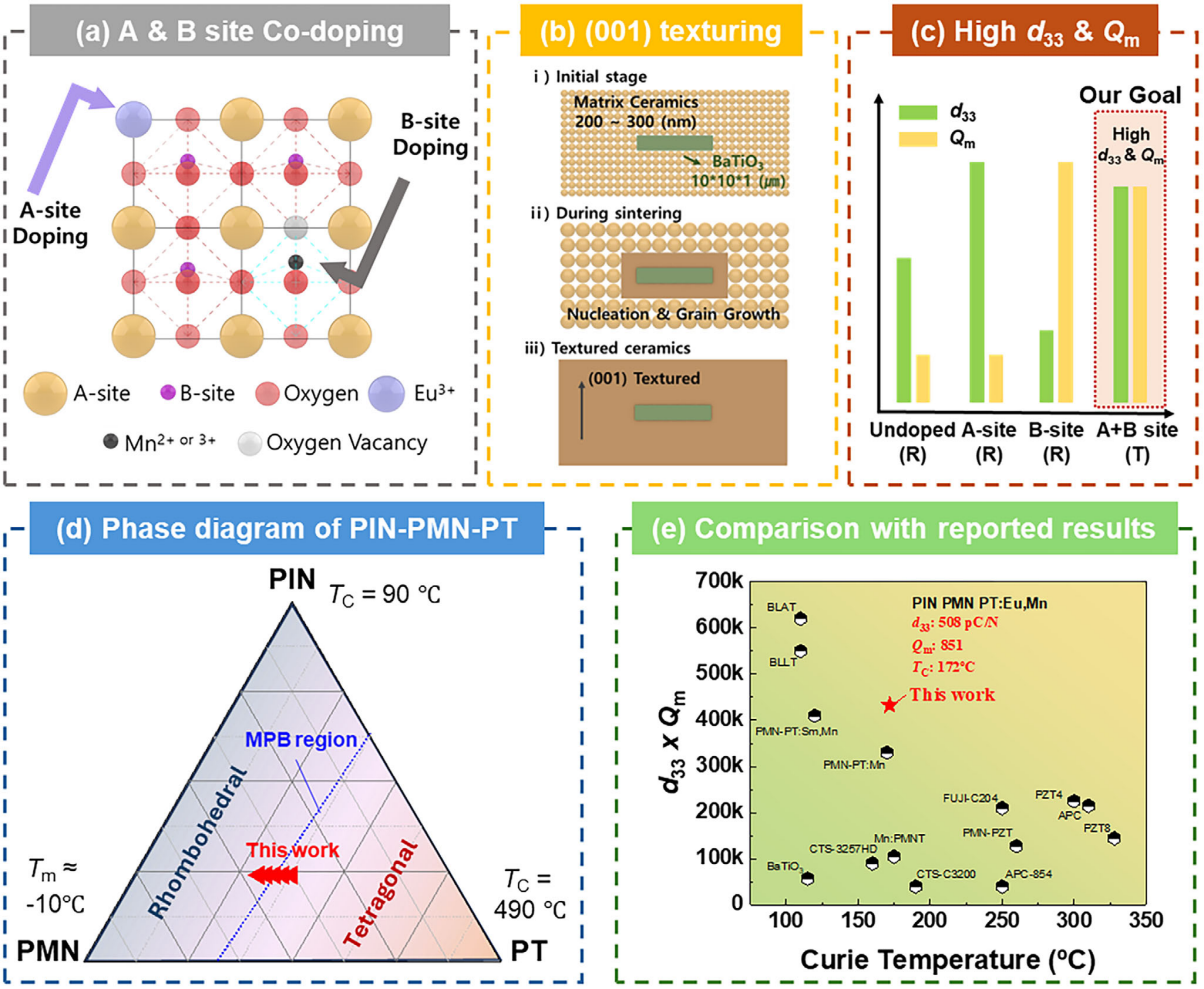
(a) Schematic illustration of the combinatorial doping strategy employing A‐site donor and B‐site acceptor ions. (b) (001) Texturing via the Template Grain Growth (TGG) process. (c) This approach enables defect and microstructure control, leading to simultaneous enhancement of the piezoelectric coefficient (*d*_33_) and mechanical quality factor (*Q*_m_) in high‐power piezoelectric ceramics. (d) Phase diagram of the PIN–PMN–PT ceramic system, highlighting the morphotropic phase boundary (MPB, blue line). (e) Comparison of Curie temperature and transducer figure of merit (*Q*_m_ × *d*_33_) for the material developed in this study and other high‐power piezoelectric ceramics reported in the literature.

In this investigation, we synthesize these complementary approaches by systematically introducing Eu ion as an A‐site donor and Mn ion as a B‐site acceptor within the PIN‐PMN‐PT system, followed by controlled grain texturing through the TGG process, as illustrated in Figure 1a–e [30, 31, 32, 33]. This integrated materials design strategy yields piezoelectric ceramics with exceptional performance metrics: *d*_33_ values reaching 508 pC/N, *k*_p_ values achieving 64%, and *Q*_m_ exceeding 850 – all while maintaining Curie temperatures above 170°C. The resulting transducer Figure of Merit (*d*_33_ × *Q*_m_) surpasses 400,000, establishing a new benchmark for high‐power piezoelectric materials that could potentially revolutionize transducer technology. Beyond the specific composition studied here, this work demonstrates a generalizable approach to overcoming fundamental property trade‐offs in functional ceramics through strategic manipulation of defect chemistry and microstructural architecture.

## 2 Results and Discussion

To maximize piezoelectric performance, this study focused on the optimized morphotropic phase boundary (MPB) composition of 0.24PIN–0.42PMN–0.34PT, as indicated by the red marker in Figure 1d [28, 29, 33, 34]. This composition lies near the ternary MPB, which is known for superior dielectric and ferroelectric properties, along with a relatively high Curie temperature [35]. Owing to these advantages, it has been regarded as a promising candidate for transducer and actuator applications. Furthermore, this composition is well‐suited for inducing crystallographic texturing via the TGG process. In this study, compositions with varying PbTiO_3_ (PT) content centered around 0.34PT were investigated. This approach enables a systematic study of phase‐TGG compatibility, as increasing the PT ratio promotes a transition toward the tetragonal phase, whereas decreasing it favors the rhombohedral phase.

The selection of the doping site in the perovskite structure is strongly influenced by the ionic radius. Figure S1 shows the ionic radii of various dopants and their corresponding substitution sites. A gradual decrease in lattice volume is observed as the ionic radius decreases from Pb^2+^ (1.49 Å at CN = 12) to La^3+^ (1.36 Å), Nd^3+^ (1.27 Å), Sm^3+^ (1.24 Å), and Eu^3+^ (1.17 Å) [36], suggesting substitution of Pb^2+^ by these dopants at the A‐site. Eu^3+^, possessing the smallest ionic radius among typical A‐site dopants, marks the lower limit for A‐site substitution and is expected to induce greater local structural heterogeneity. Gd^3+^, Dy^3+^, and Y^3+^ dopants are likely to replace smaller B‐site cations, including Mg^2+^ (0.72 Å, CN = 6), Nb^5+^ (0.64 Å), or Ti^4+^ (0.605 Å). Therefore, the suitable ionic radius range for A‐site substitution is approximately 120–140 pm, while B‐site substitution typically occurs for ions with radii between 60 and 100 pm.

In this study, Eu ion, which has a smaller ionic radius and results in a reduced cell volume compared to commonly used Sm ion, was selected to induce enhanced local structural heterogeneity [31, 37]. For acceptor doping at the B‐site, Mn ion was selected. Although Mn is a transition metal capable of exhibiting mixed valence states (Mn^2+^ = 0.83 Å, Mn^3+^ = 0.645 Å at CN = 6) at elevated temperatures, its ionic radius is well‐suited for incorporation into the B‐site lattice. Furthermore, Mn ion is recognized as one of the most effective dopant for inducing a hardening effect in the PIN‐PMN‐PT system, making it an ideal co‐dopant alongside Eu ion in this study [21, 28].

**FIGURE 2:**
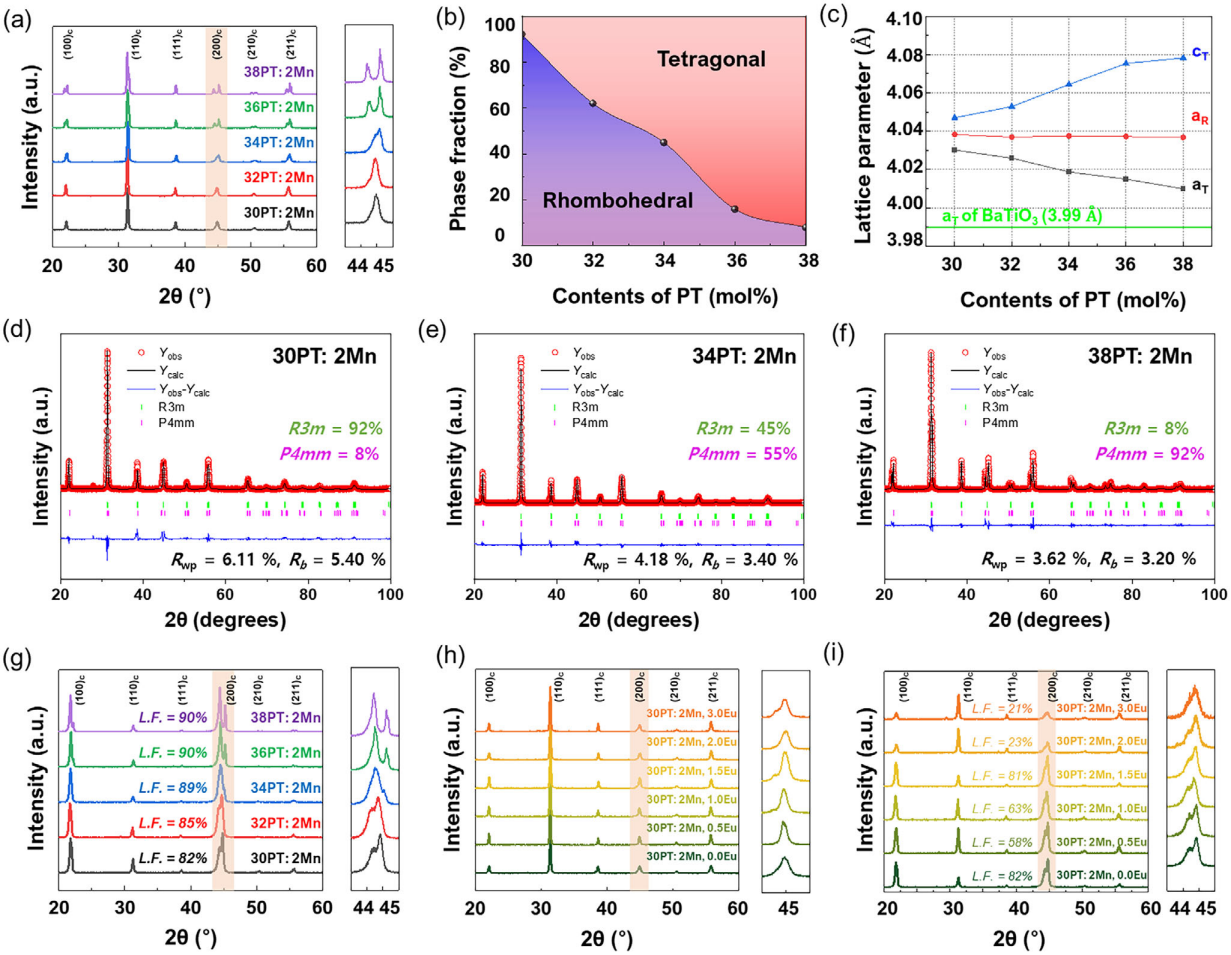
(a) XRD patterns of randomly oriented 0.24PIN–(0.76‐x)PMN–xPT (*x* = 0.30 to 0.38) ceramics with 2 mol% Mn, (b) corresponding phase fraction of rhombohedral and tetragonal phase, and (c) lattice parameter calculated from Rietveld refinement. Rietveld refinement results of randomly oriented 0.24PIN–(0.76−x)PMN–xPT (*x* = 0.30, 0.34, and 0.38) ceramics doped with 2 mol% Mn are shown in (d–f), respectively. g) XRD patterns of (001)‐oriented 0.24PIN–(0.76−x)PMN–xPT (*x* = 0.30 to 0.38) ceramics with 2 mol% Mn showing an increase in the Lotgering factor (L.F.) with PT content. h) XRD patterns of randomly oriented and (i) (001)‐oriented textured ceramics with a fixed 0.24PIN‐0.46PMN‐0.30PT composition, co‐doped with 2 mol% Mn and 0–3 mol% Eu, illustrating the effect of Eu concentration on the phase structure.

For all compositions in this study, Mn ion doping level was maintained at 2 mol%, as this concentration yielded the highest *Q*_m_ value (Figure S2), consistent with previous reports [21]. During the TGG process used to induce texturing, 1 vol% BaTiO_3_ seed were employed. While these seeds promote crystallographic texturing and enhance the *d*_33_ value, they can also act as impurities that degrade the *Q*_m_. Therefore, to optimize both *Q*_m_ and *d_33_* values, the seed content was carefully limited to 1 vol%. The XRD patterns of randomly oriented 2 mol% Mn‐doped 0.24PIN–(0.76–*x*)PMN–*x*PT ceramics (Figure 2a) reveal a gradual shift of diffraction peaks toward higher angles with increasing PT content, indicating lattice contraction. This structural evolution is consistent with the phase fraction analysis obtained from Rietveld refinement (Figure 2b), which shows a rhombohedral–tetragonal boundary in the 32–36 mol% PT range. With higher PT contents, the tetragonal fraction becomes dominant, confirming a composition‐driven phase transition. The corresponding lattice parameters (Figure 2c) further support this observation, as the separation between the a‐ and c‐axes becomes more distinct, reflecting enhanced tetragonality.

In addition, detailed Rietveld refinement was performed to quantify the phase evolution as a function of PT content in 0.24PIN–(0.76–x)PMN–xPT ceramics doped with 2 mol% Mn. As shown in Figure 2d–f, the randomly oriented specimens exhibited a gradual structural transition from a predominantly rhombohedral (R3m) symmetry at lower PT content to a tetragonal (P4mm) symmetry at higher PT ratios. Specifically, the 30PT:2Mn composition contained ∼92% R3m and ∼8% P4mm, while the fraction of P4mm increased to ∼55% at 34PT:2Mn and further to ∼92% at 38PT:2Mn, confirming a composition‐driven R–T phase evolution. To further verify this structural trend, additional Rietveld refinements for intermediate compositions (32PT and 36PT) are presented in Figure S3. The 32PT:2Mn sample exhibited a mixed‐phase structure with ∼62% R3m and ∼38% P4mm, whereas the 36PT:2Mn specimen showed a dominant tetragonal structure of ∼84% P4mm. These results support the existence of a morphotropic phase boundary (MPB). The refinement quality was evaluated using the weighted profile R‐factor (*R*_wp_) and the Bragg R‐factor (*R*_b_), which are commonly used to assess the reliability of phase fraction analysis in multiphase perovskite systems. For all refined compositions, the obtained *R*_wp_ and *R*_b_ values fall within commonly accepted ranges (below 10%).

For (001)‐textured ceramics (Figure 2g), the XRD results demonstrate that the Lotgering factor (*L.F*.) systematically increases with PT content. This behavior agrees with the lattice mismatch between the perovskite matrix and BaTiO_3_ seeds used for templated growth (Figure 2c). Since the sole addition of Eu_2_O_3_ to the system caused tetragonal‐rich phases (Figure S4), the *x* = 0.30 composition, exhibiting a more rhombohedral character, was selected for co‐doping study using MnO_2_ and Eu_2_O_3_ to induce favorable MPB characteristics. For the randomly oriented ceramics shown in Figure 2h, the effect of Eu ion content on the phase change was not significant, indicating the presence of MPB characteristics across all compositions. Figure 2i shows the XRD patterns of (001) textured 2 mol% Mn and *y* mol% Eu‐doped 0.24PIN–0.46PMN–0.30PT ceramics (*y* = 0–3). In the case of co‐doping in the *x* = 0.30 composition, a rhombohedral‐rich phase was maintained even with increasing Eu ion content. However, the optimal (001) grain alignment with Lotgering factor of 81% was achieved at 1.5 mol% Eu content. As the Eu content increased to 2 mol% and 3 mol%, the Lotgering factor dropped sharply to 23% and 21%, respectively, indicating limited (001)‐oriented grain growth near the BaTiO_3_ seeds. Excessive Eu doping at A‐site may induce cation vacancies, which accumulate at grain boundaries when their concentrations exceed critical levels. This accumulation creates a pinning effect that inhibits grain growth [31]. Consequently, as shown in Figure S5, grain growth around BaTiO_3_ seeds is significantly suppressed at higher Eu doping amounts, hindering the formation of larger grains.

**FIGURE 3:**
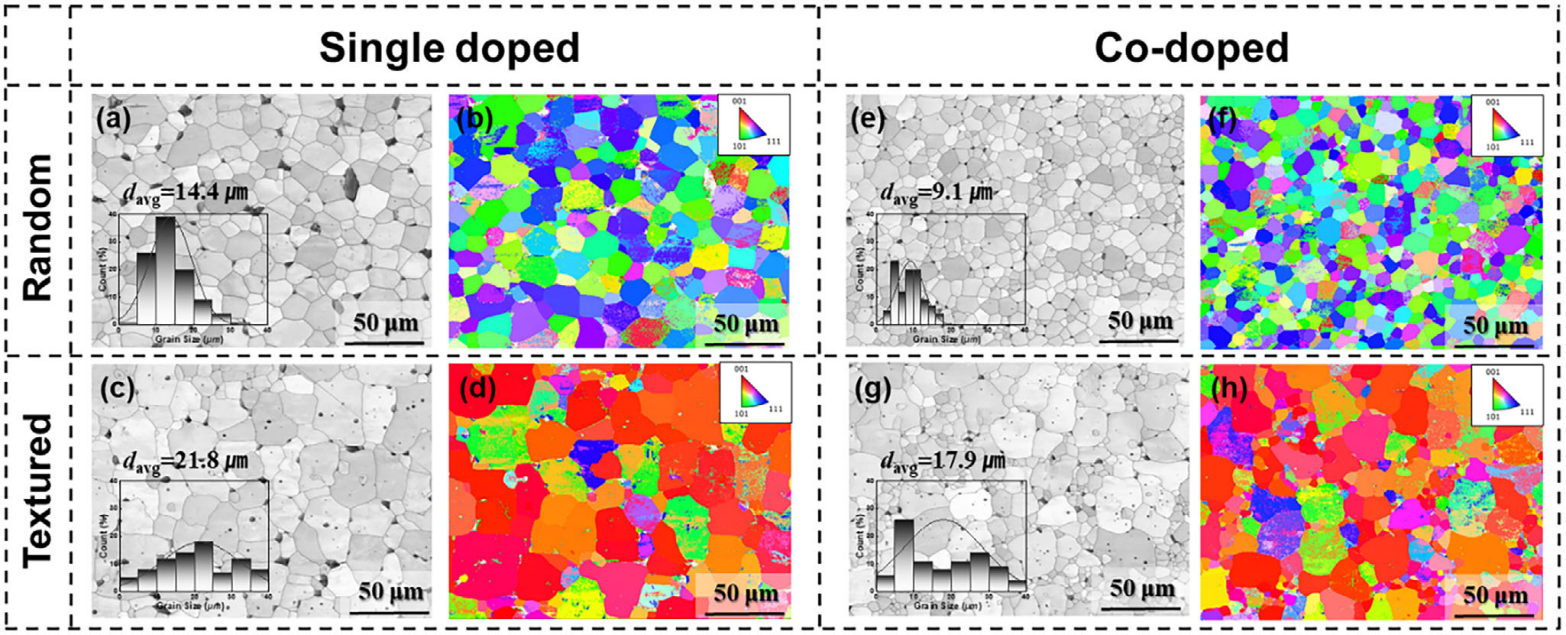
SEM and EBSD image of 0.24PIN–0.44PMN–0.32PT ceramic with 2 mol% Mn single doping and different crystallographic orientation: (a) SEM and (b) EBSD image of randomly oriented ceramic, (c) SEM and (d) EBSD image of (001)‐oriented ceramic. (e) SEM and f) EBSD image of randomly oriented 0.24PIN–0.46PMN–0.30PT ceramic co‐doped with 2 mol% Mn and 1.5 mol% Eu, (g) SEM and (h) EBSD image of (001)‐oriented 24PIN–0.46PMN–0.30PT ceramic with the same co‐doping.

Figure 3 shows the surface SEM and EBSD images of randomly oriented and (001)‐textured 0.24PIN–0.44PMN–0.32PT ceramics with 2 mol% Mn single‐doping (Figure 3a–d) and 0.24PIN–0.46PMN–0.30PT ceramics with 2 mol% Mn + 1.5 mol% Eu co‐doping (Figure 3e–h). All ceramic samples exhibited dense microstructures with relative densities exceeding 90%. In both the single‐doped and co‐doped cases, the textured specimens show an increase in grain size compared to the randomly oriented specimens, indicating effective (001)‐textured grain growth (TGG) on 1 vol% BT seeds. For both randomly oriented and textured specimens, the grain size in the co‐doped samples is smaller than that in the single‐doped samples. In the PIN‐PMN‐PT system, Mn doping induces the formation of a liquid phase and oxygen vacancies, which enhance grain boundary mobility and lead to an increase in grain size compared to pure PIN‐PMN‐PT [38]. However, A‐site Pb vacancies introduced by Eu co‐doping, unlike oxygen vacancies, tend to accumulate near the grain boundaries. This accumulation can induce lattice distortion and destabilize the grain boundaries, thereby reducing their mobility and acting as grain boundary pinning centers [31]. Therefore, the smaller grain size in the co‐doped specimens is attributed to this effect. Furthermore, as observed in the surface EBSD analysis (Figure 3d,h), both single‐doped and co‐doped (001) textured specimens show (001) orientation at the surface compared to the randomly oriented specimens, confirming that the alignment of BT seeds within the specimens was successfully achieved.

**FIGURE 4:**
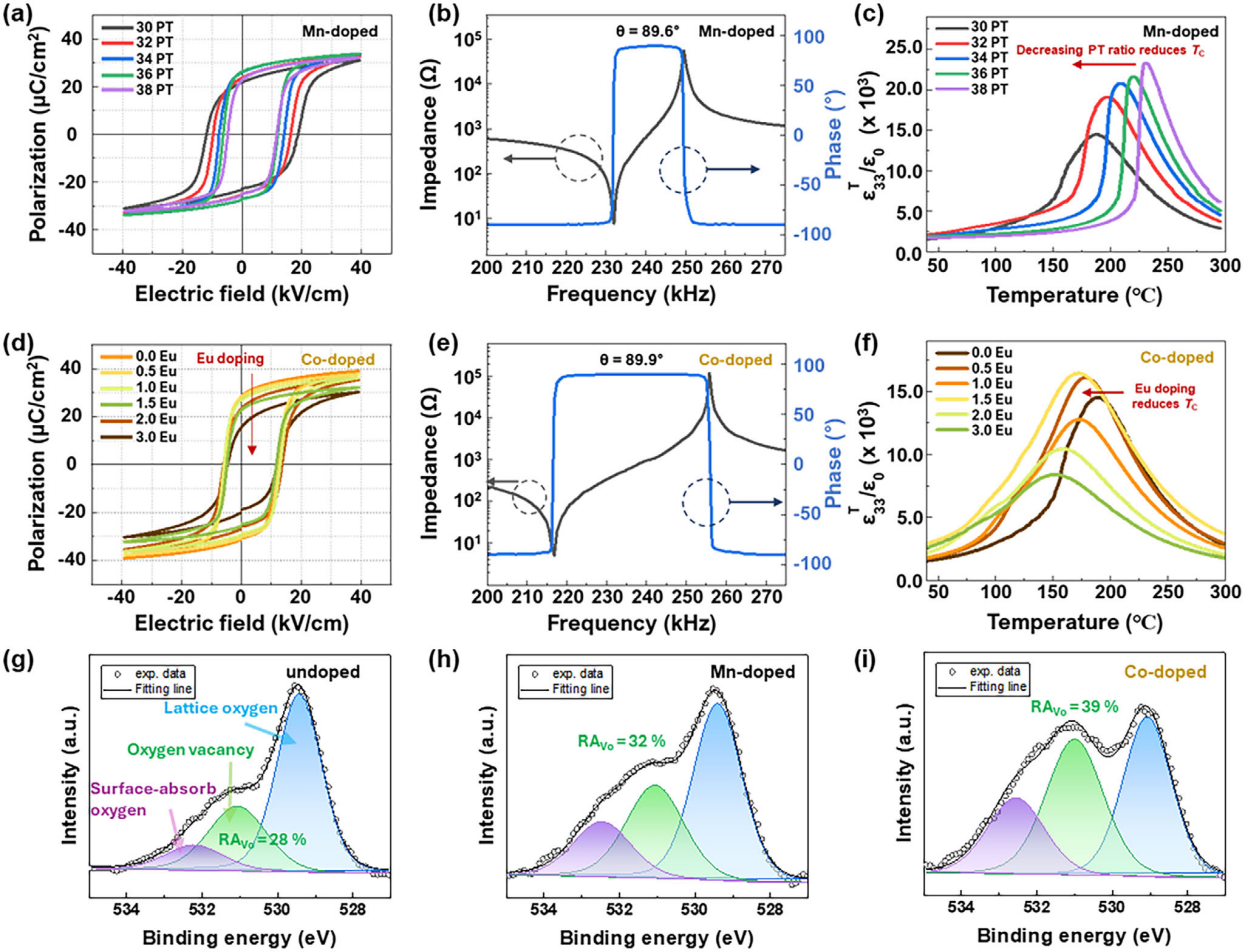
(a) Polarization–electric field (P–E) hysteresis loops of 2 mol% Mn‐doped (001)‐oriented 0.24PIN‐(0.76‐x)PMN‐xPT ceramics with PT compositions ranging from 0.30 to 0.38. (b) Impedance and phase angle spectra of 2 mol% Mn‐doped (001)‐oriented 0.24PIN–0.44PMN–0.32PT textured ceramics. (c) Temperature‐dependent dielectric permittivity of 2 mol% Mn‐doped (001)‐oriented 0.24PIN‐(0.76‐x)PMN‐xPT across the PT composition range from 0.30 to 0.38. (d) P–E hysteresis loops of Mn 2 mol% and Eu 0 to 3 mol% co‐doped (001)‐oriented ceramics at the 0.24PIN‐0.46PMN‐0.30PT composition. (e) Impedance and phase angle spectra of Mn 2 mol% and Eu 1.5 mol% co‐doped (001)‐oriented ceramics at the 0.24PIN‐0.46PMN‐0.30PT composition. (f) Temperature‐dependent dielectric permittivity of Mn 2mol% and Eu 0 to 3 mol% co‐doped (001)‐oriented 0.24PIN–0.46PMN–0.30PT textured ceramics. XPS O 1s spectra, deconvoluted into lattice oxygen, oxygen vacancy, and surface‐absorbed oxygen components, of (g) undoped 0.24PIN–0.44PMN–0.32PT ceramics, (h) Mn‐doped 0.24PIN–0.44PMN–0.32PT ceramics, and (i) co‐doped 0.24PIN–0.46PMN–0.30PT samples.

Figure 4 presents the ferroelectric and dielectric properties of (001)‐textured 0.24PIN–(0.76–x)PMN–xPT ceramics with 2 mol% Mn single‐doping ((001)‐2Mn–xPT, x = 0.30–0.38) and (001)‐textured 0.24PIN–0.46PMN–0.30PT ceramics with 2 mol% Mn and y mol% Eu co‐doping ((001)‐2Mn/yEu–0.30PT, y = 0–3). As shown in Figure 4a, all Mn‐doped textured specimens exhibited pronounced asymmetry between the positive and negative coercive fields (*E*_c_) in the polarization–electric field (P–E) hysteresis loops. This asymmetric switching behavior indicates the presence of a strong internal bias, originating from defect dipoles associated with oxygen vacancies generated through Mn doping. Figure 4b shows the impedance and phase angle spectra of the Mn‐doped 0.32PT specimen. The sharp resonance and anti‐resonance peaks and rectangular phase‐angle profile indicate low mechanical loss and well‐developed electromechanical coupling. The temperature‐dependent dielectric permittivity in Figure 4c shows that decreasing PT content lowers the Curie temperature (*T*_C_) and broadens the dielectric peak. This trend reflects the relative contributions of PT (a normal ferroelectric with high *T*_C_) and PMN/PIN (relaxor ferroelectrics with low *T*_C_ and *T*_m_).

Figure 4d displays the P–E behavior of Mn/Eu co‐doped textured ceramics at the fixed 0.30PT composition. Unlike the Mn‐doped series, *E*_c_ remains nearly unchanged with Eu content, whereas *P*_r_ decreases with Eu content significantly. This decline corresponds to the A‐site vacancy formation during Eu doping. As shown in Figure 4e, the co‐doped specimen (y = 1.5) exhibits a larger frequency separation between resonance and anti‐resonance peaks compared to the Mn‐doped specimen. This behavior indicates stronger electromechanical coupling. Together with the P–E trends, this confirms that Mn/Eu co‐doping induces both hardening (from Mn) and softening (from Eu), likely due to spatial separation of A‐site donors and B‐site acceptors. The depolarization behavior was examined by temperature‐dependent P–E measurements (Figure S6). Both Mn‐doped and Mn/Eu co‐doped ceramics show stable ferroelectric hysteresis well below the Curie temperature, followed by a rapid collapse of polarization near *T*_C_. Figure 4f presents the dielectric permittivity of Mn/Eu co‐doped ceramics. Increasing Eu content broadens the dielectric peak, reflecting enhanced relaxor‐like behavior. This suppression of ergodicity helps maintain favorable ferroelectric and piezoelectric performance.

Figure 4g–i shows the O 1s XPS spectra of undoped, Mn‐doped, and Mn/Eu co‐doped PIN–PMN–PT ceramics, deconvoluted into lattice oxygen, oxygen‐vacancy, and surface‐adsorbed oxygen. The undoped ceramic exhibits a dominant lattice oxygen contribution, while Mn‐doped ceramics show a pronounced increase in the oxygen vacancy peak, consistent with the formation of Mn–oxygen vacancy defect dipoles responsible for the hardening effect. Here, RA_Vo_ is defined as the relative area fraction of the oxygen‐vacancy O 1s component obtained from peak deconvolution, expressed as a percentage of the total O 1s signal. The RA_Vo_ value increases from ∼28% in the undoped ceramic to ∼32% in the Mn‐doped sample, consistent with the formation of oxygen vacancy induced by B‐site Mn acceptor doping. The Mn/Eu co‐doped ceramic exhibits a relatively higher RA_Vo_ value (∼39%), however, this increase should not be directly attributed to the Eu donor effect alone. It should be noted that the Mn‐doped and co‐doped samples have different PT contents (0.32PT and 0.30PT, respectively). Therefore, a direct quantitative comparison of RA_Vo_ values is not appropriate. Instead, the XPS results qualitatively indicate that oxygen‐vacancy‐related functional defects are preserved in the co‐doped system without being suppressed by donor–acceptor recombination, supporting the proposed synergistic co‐doping mechanism.

**FIGURE 5:**
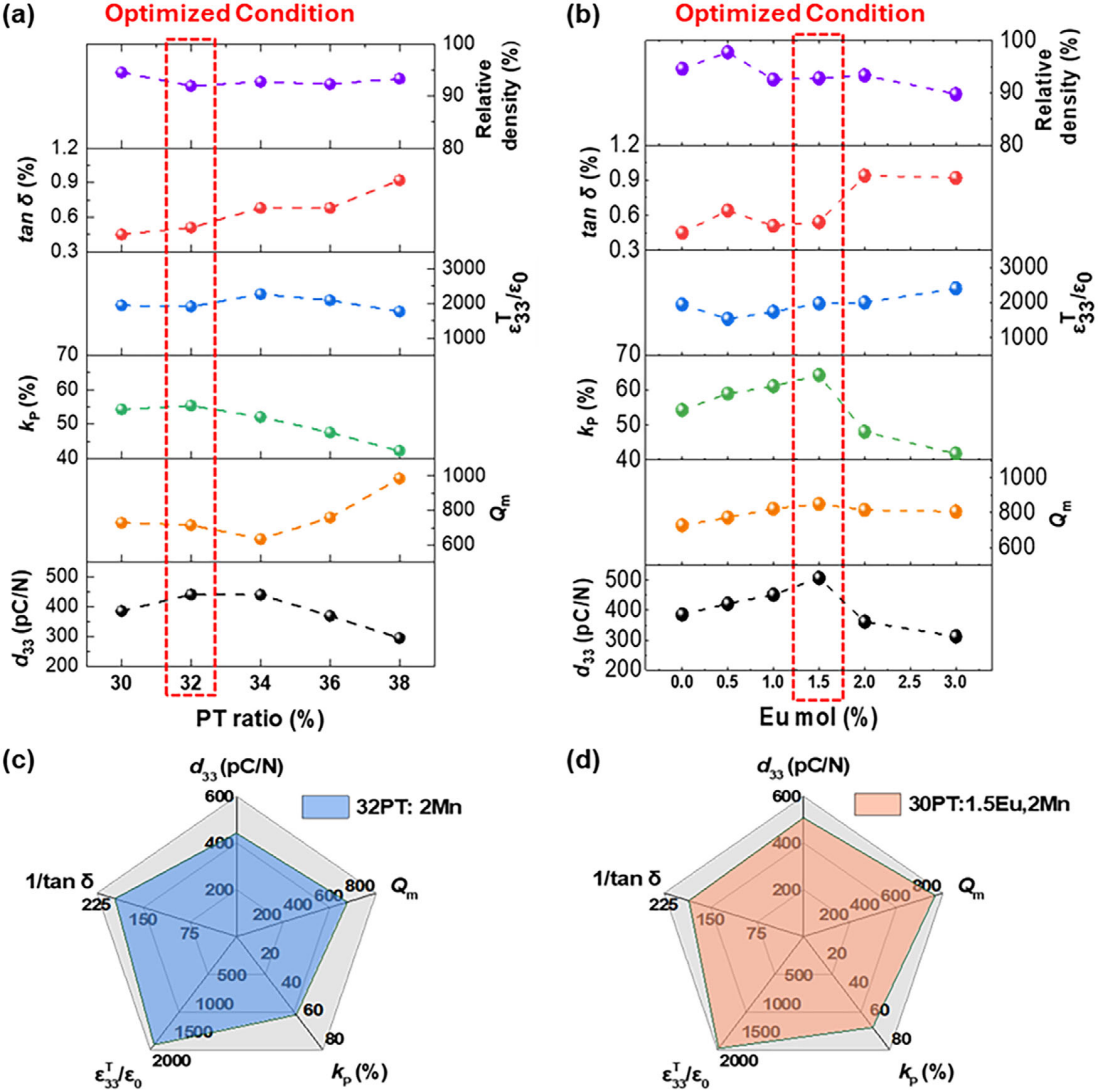
(a) Dielectric and piezoelectric properties of (001)‐oriented textured 2 mol% Mn single‐doped 0.24PIN–(0.76–*x*)PMN–*x*PT ceramics with PT compositions ranging from 0.30 to 0.38. (b) Dielectric and piezoelectric properties of 2 mol% Mn and *y* mol% Eu co‐doped 0.24PIN–0.46PMN–0.30PT ceramics with Eu content varying from 0 to 3 mol%. Spider charts of 1/tan δ, $\;\varepsilon_{33}^{T}/\varepsilon_{0}$, *k*_p_, *Q*_m_ and *d*_33_ for (c) 2 mol% Mn single‐doped 0.24PIN‐0.44PMN‐0.32PT textured ceramics, and (d) 2 mol% Mn and 1.5 mol% Eu co‐doped 0.24PIN‐0.46PMN‐0.30PT textured ceramics.

Figure 5 presents a comprehensive comparison of the piezoelectric and dielectric performance of the (001)‐2Mn‐*x*PT and (001)‐2Mn/*y*Eu‐0.30PT specimens. Among the (001)‐2Mn‐*x*PT samples, the *x* = 0.32 composition, identified as a morphotropic phase boundary (MPB) composition, exhibited the highest soft piezoelectric properties, with *d*_33_ = 442 pC/N and *k*_p_ = 55.4%, as shown in Figure 5a. For the (001)‐2Mn/*y*Eu‐0.30PT specimens (Figure 5b), the soft piezoelectric properties gradually improved with increasing *y*, reaching significantly enhanced values of *d*_33_ = 508 pC/N and *k*_p_ = 64.3% at *y* = 1.5. This improvement can be attributed to the development of piezoelectric anisotropy caused by TGG with a high Lotgering factor, as well as the increased presence of a non‐ergodic polar phase induced by appropriate Eu doping. Despite the enhanced soft piezoelectricity, the specimen with *y* = 1.5 maintained a high *Q*_m_ value of 851, demonstrating that the combination of Mn and Eu co‐doping with (001)‐texturing can simultaneously induce both hardening and softening effects. This result experimentally confirms that such a strategy is a promising approach for achieving both high *d*_33_ and high *Q*_m_ in piezoelectric materials. As illustrated in the spider charts in Figure 5c,d, the (001)‐2Mn/1.5Eu‐0.30PT specimen exhibits superior overall piezoelectric and dielectric properties compared to the (001)‐2Mn‐0.32PT specimen. This noticeable enhancement in overall electrical performance, including *d*_33_, *Q*_m_, and *k*_p_, further underscores that the synergistic co‐doping of Eu and Mn plays a critical role in enhancing the piezoelectric response.

**FIGURE 6:**
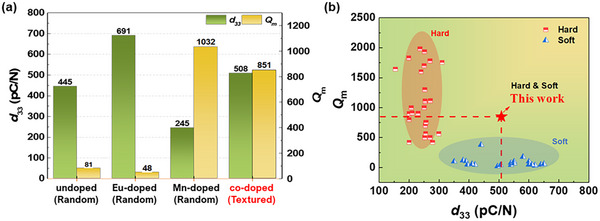
(a) Piezoelectric coefficient (*d*_33_) and mechanical quality factor (*Q*_m_) of 0.24PIN–(0.76‐x)PMN–xPT ceramics with varying doping strategies: undoped, A‐site donor doped, B‐site acceptor doped, and combinatorially co‐doped at both A‐ and B‐sites with TGG texturing. (b) Comparison of *d*_33_ and *Q*_m_ values obtained in this work with those of representative hard and soft piezoelectric ceramics.

The temperature‐dependent piezoelectric and dielectric properties of the textured ceramics were systematically investigated to clarify their high‐temperature behavior (Figures S7 and S8). For the Mn single‐doped textured ceramics, both *d*_33_ and *k*_p_ exhibit relatively stable values with increasing temperature up to approximately 200°C, close to the Curie temperature. This behavior indicates that the intrinsic piezoelectric response is well maintained below *T*_C_. In contrast, the mechanical quality factor *Q*_m_ of the Mn‐doped ceramics shows a gradual, nearly linear decrease with increasing temperature. This behavior closely correlates with the temperature dependence of the dielectric loss, which increases almost linearly at lower temperatures and rises sharply above ∼180°C. For the Mn/Eu co‐doped textured ceramics, both *d*_33_ and *k*_p_ are also well maintained over a wide temperature range below *T*_C_, below ∼150°C. Once the temperature exceeds *T*_C_, the piezoelectric response vanishes, consistent with the ferroelectric–paraelectric phase transition. The *Q*_m_ values of co‐doped ceramics continuously decrease with temperature and exhibit an inverse relationship with tan δ, like the Mn single‐doped case. Figure 6a presents a comparison of *d_33_* and *Q_m_* values for different doping configurations in textured 0.24PIN‐0.46PMN‐0.30PT ceramics. The undoped ceramic exhibited a “soft” characteristic with a *d*_33_ value of 445 pC/N and a *Q*_m_ of 81. When Eu was singly doped at the A‐site at a concentration of 1.5 mol%, the enhanced piezoelectric properties resulted in a maximum *d*_33_ of 691 pC/N. In contrast, 2 mol% Mn single doping at the B‐site exhibited a pronounced hardening effect, where *d*_33_ decreased to 245 pC/N, but *Q*_m_ significantly increased from 81 to 1032. When both A‐site and B‐site were doped simultaneously and grain were textured through TGG process ((001)‐2Mn/1.5Eu‐0.30PT), the ceramic achieved an optimal balance with *d*_33_ = 508 pC/N and *Q*_m_ = 851, demonstrating improvements in both soft and hard properties. Figure 6b compares the properties of the (001)‐2Mn/1.5Eu‐0.30PT ceramic developed in this study with those of previously reported soft and hard piezoelectric ceramics. The (001)‐2Mn/1.5Eu‐0.30PT ceramic exhibits a unique combination of soft and hard characteristics, simultaneously achieving high *d*_33_ and *Q*_m_ values, thereby demonstrating superior performance compared to most commercially available and previously reported piezoelectric materials [2]. Furthermore the (001)‐2Mn/1.5Eu‐0.30PT ceramic fabricated in this study achieve a transducer Figure‐of‐Merit (FOM), defined as *d*_33_ × *Q*_m_, exceeding 400 000—surpassing the performance of recently reported materials with high Curie temperatures (Figure 1e) [38, 39]. A comprehensive comparison of the dielectric, piezoelectric, and electromechanical parameters of the present ceramics, together with representative commercial hard piezoelectric materials, is summarized in Table 1. This highlights their exceptional performance and suitability for high‐power applications.

**TABLE 1:** $\varepsilon_{33}^{T}/\varepsilon_{0}$, *d*_33_ coefficient, mechanical quality factor (*Q*_m_), electromechanical coupling factor (*k*_p_), Curie temperature (𝑇_C_), and *d*_33_ * *Q*_m_ for the Pb based hard piezoelectric ceramics.

Composition	$\varepsilon_{33}^{T}$ */* $\varepsilon_{0}^{1}$	*d*_33_ (pC/N)	*Q*_m_	*k*_p_ (%)	*T*_c_ (°C)	tan δ (%)	*d*_33_ *Q_m_ (10^3^ pC/N)	Refs.
(001) Mn‐doped PIN‐PMN‐PT	1912	442	715	55	200	0.51	316	This work
(001) Mn/Eu co‐doped PIN‐PMN‐PT	1977	508	851	64	170	0.54	432	This work
Commercial Hard PZT (PZT4)	1300	290	500	61	328	0.3	145	[40]
Commercial Hard PZT (PZT8)	1000	225	1000	57	300	0.5	225	[40]

## 3 Conclusion

This study successfully developed high‐power piezoelectric ceramics with enhanced temperature stability by employing the templated grain growth (TGG) technique alongside combinatorial co‐doping of A‐site donors and B‐site acceptors in PIN–PMN–PT compositions. High‐power applications demand materials with excellent thermal stability to withstand the heat generated by mechanical losses at high frequencies, as excessive heat can significantly degrade performance. By incorporating constituents with high Curie temperatures‐PIN (*T*_C_ = 90°C), PMN (*T*_m_ = −10°C), and PT (*T*_C_ = 490°C)‐into the ternary system, the overall Curie temperature was maintained above 200°C, enabling reliable operation in high‐power environments. In this work, donor and acceptor dopants were systematically optimized to simultaneously enhance both the piezoelectric coefficient (*d*_33_) and mechanical quality factor (*Q*_m_). Eu_2_O_3_ was introduced at the A‐site, while MnO_2_ was incorporated at the B‐site. Eu doping led to contraction along the a‐ and b‐axes, generating local structural heterogeneity that contributed to enhanced d_33_. In contrast, MnO_2_ induced a domain pinning effect, suppressing domain wall motion and improving *Q*_m_. Furthermore, spatial separation of donor and acceptor doping between the A and B‐sites minimized the recombination of cation and oxygen vacancies, thereby reducing defect‐related degradation. The optimized co‐doping concentrations of 2 mol% Mn and 1.5 mol% Eu, in combination with the TGG process, yielded outstanding electromechanical properties: *d*_33_> 500 pC/N, *Q*_m_ > 850, *T*_C_ > 170° and *d*_33_ × *Q*_m_ > 400,000. These results mark a significant advancement in the development of high‐power piezoelectric ceramics. The co‐doped, textured PIN–PMN–PT ceramics demonstrated in this study exhibit exceptional performance and hold strong potential for integration into next‐generation high‐power piezoelectric devices.

## 4 Experimental

### 4.1 Synthesis of Randomly Oriented PIN‐PMN‐PT Ceramics

The Mn‐doped Pb(In_1/2_Nb1_1/2_)O_3_‐Pb(Mg_1/3_Nb_2/3_)O_3_‐PbTiO_3_ (PIN‐PMN‐PT) randomly oriented ceramics were synthesized using a two‐step columbite method to ensure high compositional homogeneity and minimize secondary phase formation. Initially, MgNb_2_O_6_ was synthesized to inhibit the formation of the pyrochlore phase, which easily forms due to the interaction of Mg and Nb with Pb. MgO (≥99%, Sigma‐Aldrich), being highly sensitive to moisture, was heat‐treated at 1000°C for 2 h immediately after batching to maintain accuracy. MgO and Nb_2_O_5_ (99.9%, High Purity Chemicals) were weighed in stoichiometric proportions and ball‐milled in ethanol using a Nalgene bottle with zirconia balls for 24 h. The resulting mixture was calcined at 1100°C for 4 h to produce MgNb_2_O_6_. Concurrently, In_2_O_3_ (99.9%, Thermo Scientific) and Nb_2_O_5_ powders were mixed and sintered at 1100°C for 7 h to prepare InNbO_4_ precursors. The final composition was prepared by mixing PbO (99.99%, High Purity Chemicals), MgNb_2_O_6_, InNbO_4_, TiO_2_ (99.8%, Sigma–Aldrich), MnO_2_ (≥99%, Sigma–Aldrich), and Eu_2_O_3_(≥99.9%, Sigma–Aldrich) in stoichiometric proportions. Excess PbO was added to facilitate liquid phase formation during sintering. The raw materials were ball‐milled in ethanol for 24 h and subsequently dried and calcined at 850°C for 4 h. After calcination, the powders were ground to reduce particle size, and 5 vol% polyvinyl alcohol (PVA) binder was added to facilitate shape forming. The mixture was sieved to ensure uniform granularity, and approximately 0.7 g of the powder was uniaxially pressed at 20 MPa into 12 mm‐diameter pellet molds. The PVA binder was removed by heating at 600°C for 2 h, followed by sintering at 1150°C for 2 h in a sealed alumina crucible to minimize PbO evaporation. The sintered samples were polished with SiC paper to meet the IEEE standards for piezoelectric measurements.

### 4.2 Synthesis of (001)‐Oriented PIN‐PMN‐PT Ceramics via TGG Process

The (001)‐oriented textured ceramics were fabricated using the Templated Grain Growth (TGG) process, incorporating BaTiO_3_ platelet seeds to induce preferential grain orientation. The BaTiO_3_ template seeds were synthesized via a molten salt method, ensuring controlled platelet morphology to facilitate grain texturing.

For the tape‐casting process, a stable slurry was prepared by dispersing BaTiO_3_ powder, toluene, ethanol, dispersant (BYK‐111), and binder. The slurry underwent 24‐h ball milling to ensure homogeneity. Subsequently, a binder was added at a 35 vol% powder‐to‐binder ratio. To introduce texturing effects, 0–3 vol% BaTiO_3_ seeds were incorporated into the slurry, followed by an additional 2‐h milling at 30 rpm to ensure uniform dispersion. Due to the high solvent content in the slurry, a vacuum stirring defoamer was employed to remove excess solvents and optimize viscosity for tape casting. Once the slurry viscosity reached 3000 cps, planetary milling was performed at 800 rpm for 10 min. Tape casting was carried out using a comma‐blade technique, with the following parameters: blade height of 250 µm, casting speed of 0.4 m/min, and final sheet thickness ranging from 45 to 50 µm. The drying process was conducted in two stages: 35°C in the first stage and 65°C in the second stage. As the slurry passed through the comma blade, the shear stress induced (001) orientation of the BaTiO3_3_ template seeds, promoting grain alignment during the subsequent sintering process.

Following tape casting, the green sheets were dried at room temperature for 24 h and cut into 10 × 10 cm pieces. To achieve a total thickness of 1.5 mm, multiple layers were stacked and laminated using hot pressing at 20 MPa for 30 min at 65°C. The laminated tapes were then laser‐cut into 12 mm‐diameter discs, and a burnout process was conducted to remove organic components. The burnout cycle was set at a heating rate of 0.3°C/min, maintaining 330°C for 12 h and 550°C for 3 h. To enhance densification, the samples were vacuum sealed in latex and subjected to Cold Isostatic Pressing (CIP) at 200 MPa for 20 min. The final sintering was performed in an air atmosphere at temperatures ranging from 1100°C to 1250°C for 2 to 10 h to optimize grain growth and alignment. After sintering, the samples were polished using SiC polishing paper to meet IEEE standards for piezoelectric characterization, ensuring a thickness‐to‐diameter ratio of 15:1.

### 4.3 Characterization of Piezoelectric Properties

To prepare the samples for electrical characterization, the polished specimens were cleaned in an ultrasonic bath for 5 min to remove residual particles. Silver electrodes were screen‐printed onto both surfaces of the pellets, followed by firing at 600°C for 10 min. The samples were poled in an oil bath using a DC power supply at a field strength of 40 kV/cm for 30 min. The piezoelectric properties of the samples were evaluated 24 h after the poling process. The piezoelectric coefficient (𝑑_33_) was measured using a quasi‐static 𝑑_33_ meter (Institute of Acoustics, China). Impedance spectra and phase angles were obtained using an impedance analyzer (4294A, Agilent Technologies, USA). The planar electromechanical coupling factor (*k*_p_) and mechanical quality factor (*Q*_m_) were calculated from the impedance spectra measured in the planar (radial) mode. The *k*_p_ value was obtained using *k_p_* =  [0.395(*f_r_*/(*f_a_* − *f_r_*)) + 0.574]^−1/2^, where the constants are geometry factors for the planar mode. The *Q*_m_ value was evaluated using an impedance‐equivalent circuit approach as $Q_{m}={\left[2\pi f_{r}CR\left(1-f_{r}^{2}/f_{a}^{2}\right)\right]}^{-1}$, where C is the clamped capacitance and R is the motional resistance at resonance. Ferroelectric hysteresis loops were recorded at room temperature under a 1 Hz frequency using a ferroelectric test system (Precision Premier II, Radiant Technologies Inc., USA) equipped with a Sawyer‐Tower circuit. The crystallographic structure of the samples was analyzed using X‐ray diffraction (XRD, D8 Advance, Bruker, USA) with Cu Kα radiation. The perovskite phase was scanned in the 20° to 60° range with a step size of 0.02° and a scanning speed of 8°/min. For detailed crystallographic analysis, slower scans were performed with a step size of 0.02° and a scanning rate of 0.1°/min. The actual densities of the sintered specimens were determined using the Archimedes method, while theoretical densities were calculated from XRD patterns. Microstructural observations were conducted using Scanning Electron Microscopy (SEM, Inspect F50, FEI, USA) in combination with Energy Dispersive Spectroscopy (EDS) for compositional analysis. Grain sizes were determined using Image J software, and the chemical states of oxygen in the samples were investigated using X‐ray Photoelectron Spectroscopy (XPS, Nexsa, Thermo Fisher Scientific, USA).

## Conflicts of Interest

The authors declare no conflicts of interest.

## Declaration of Generative AI and AI‐Assisted Technologies in the Writing Process

During the preparation of this work the author(s) used ChatGPT5 in order to improve language and readability. After using this tool/service, the authors reviewed and edited the content as needed and took full responsibility for the content of the published article.

## Supporting information




**Supporting File**: smtd70618‐sup‐0001‐SuppMat.docx.

## Data Availability

The data that support the findings of this study are available from the corresponding author upon reasonable request.
