# Supplementary material for: High‐Power Performance of Textured Piezoelectric Ceramics Through Synergistic A‐Site Donor and B‐Site Acceptor Doping
Source: Small Methods. 2026 Apr 1;10(9):e02326. doi: 10.1002/smtd.202502326 (PMC13159426; doi:10.1002/smtd.202502326)
Supplement: Supplementary file 1 — Supporting File: smtd70618‐sup‐0001‐SuppMat.docx. [file SMTD-10-e02326-s001.docx]

Supporting Information

High-Power Performance of Textured Piezoelectric Ceramics through Synergistic A-site Donor and B-site Acceptor Doping

Minwoo Kim^+^, Dong-Gyu Lee^+^, Il-Ryeol Yoo, Byeong-Jae Min, Ye Rok Choi, Hyun Soo Kim, Sunghoon Hur, Heemin Kang, Sahn Nahm, Jungho Ryu, Yongke Yan, Jeong Min Baik, Kyung-Hoon Cho*, and Hyun-Cheol Song*

**Supplemental Materials**

**
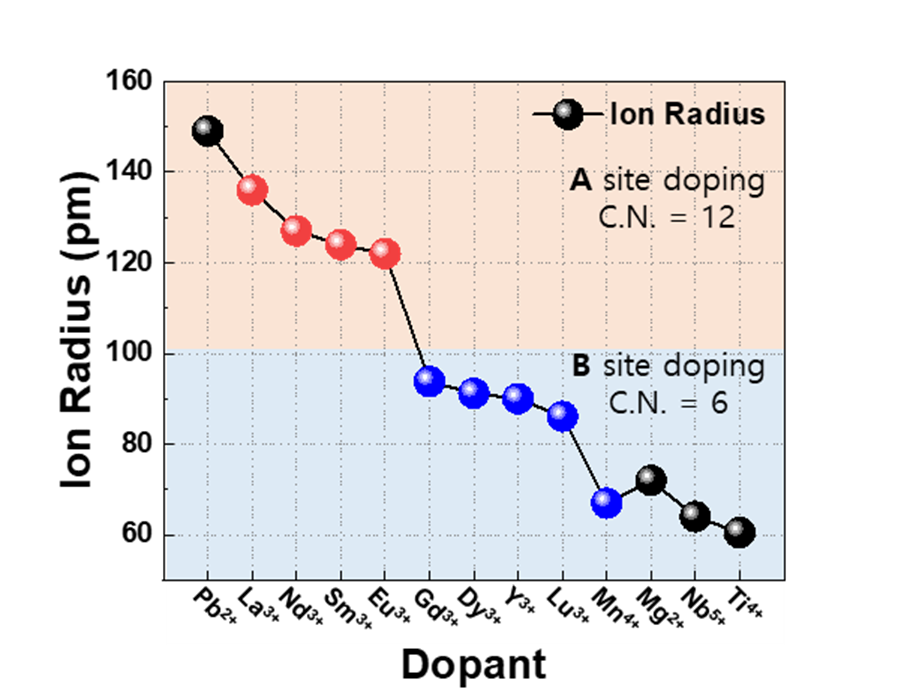
**

**Figure S1.** Comparison of the ionic radii of representative dopants for A-site and B-site substitution in the perovskite structure, presented as a function of coordination number (C.N.).


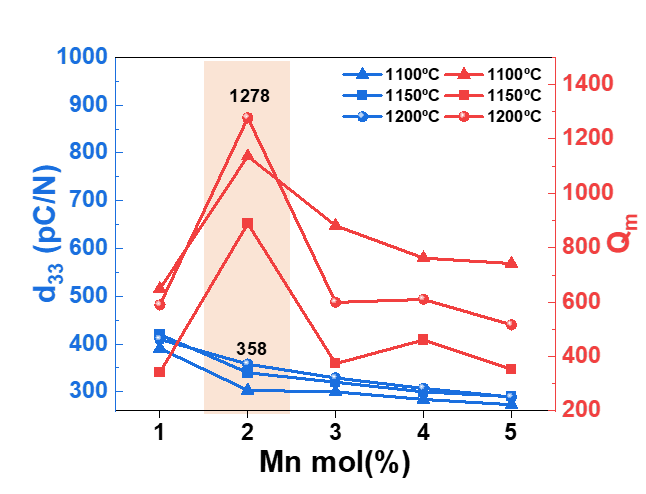


**Figure S2.** Piezoelectric coefficient (*d*₃₃) and mechanical quality factor (*Q*ₘ) of 0.24PIN-0.46PMN-0.30PT ceramics doped with *z* mol% Mn (*z* = 1–5) as a function of sintering temperature.


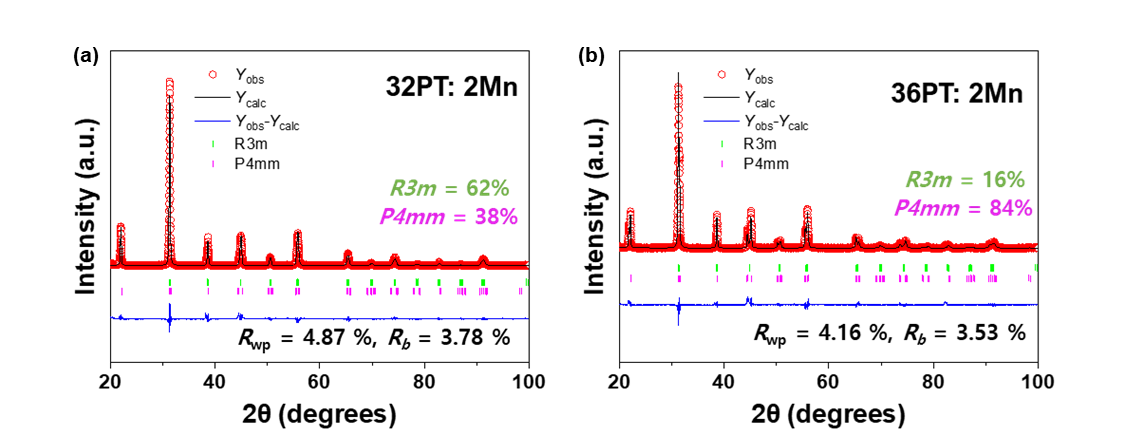


**Figure S3.** Rietveld refinement results of randomly oriented 0.24PIN–(0.76-x)PMN–xPT (x = 0.32 and 0.36) doped with 2 mol% Mn are shown in a) and b).


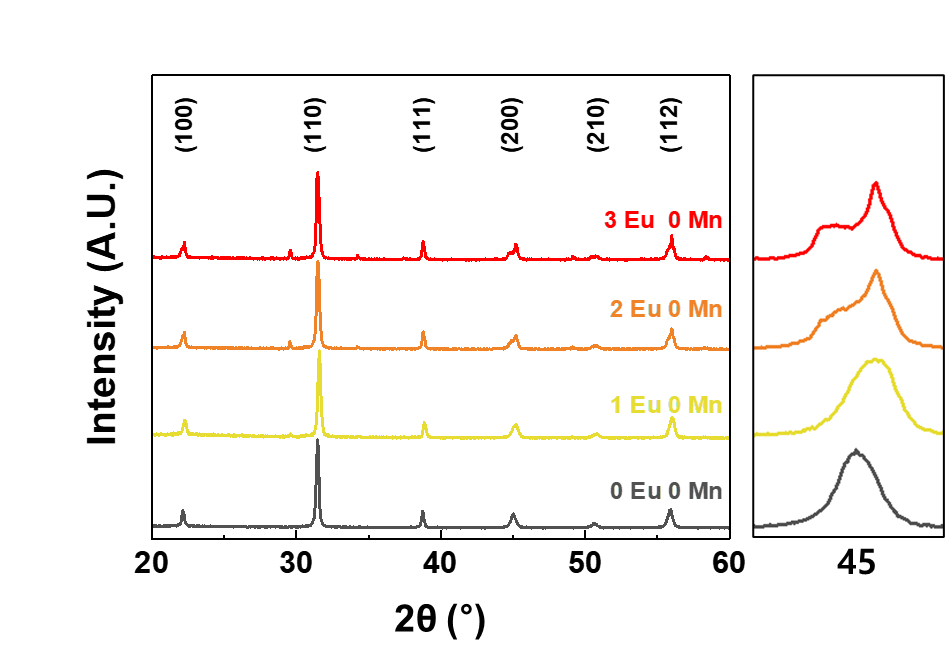


**Figure S4.** XRD patterns of textured 0.24PIN–0.46PMN–0.30PT ceramics with A-site Eu doping at varying concentrations (*y* = 0–3 mol%).


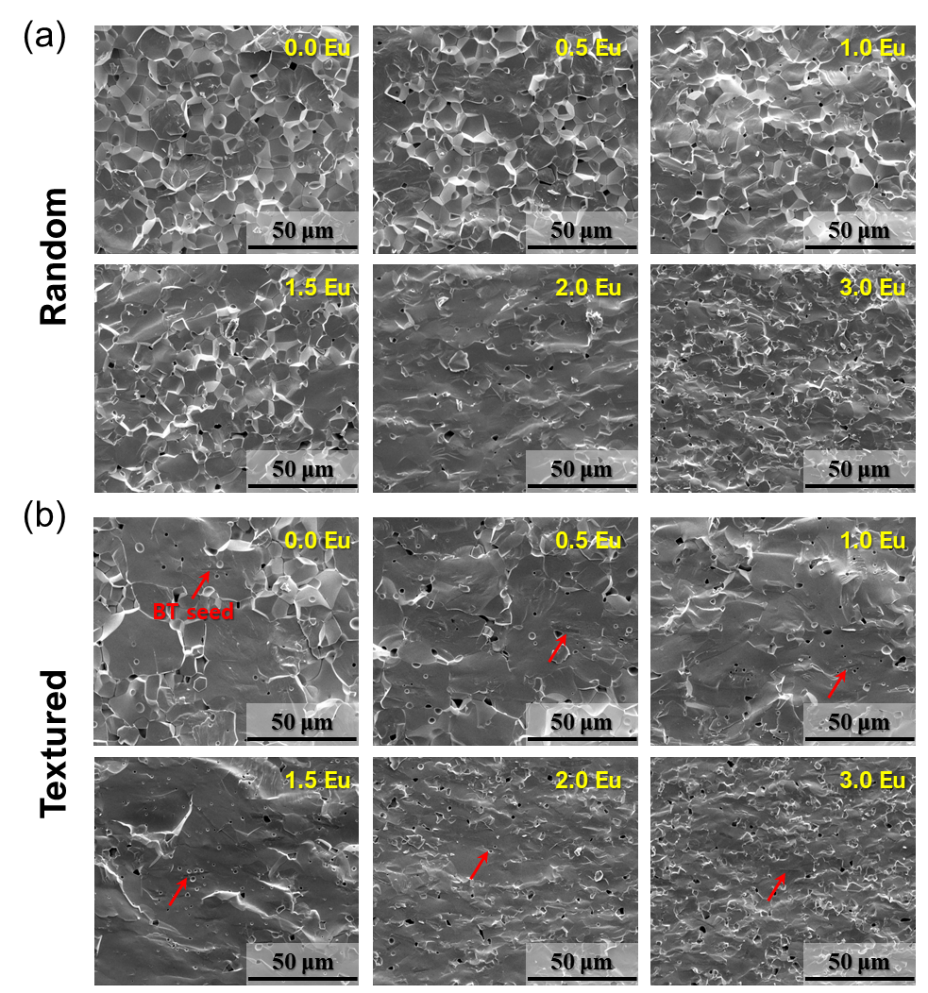


**Figure S5.** Cross-sectional SEM images of 0.24PIN–0.42PMN–0.30PT ceramics co-doped with 2 mol% Mn and *y* mol% Eu (*y* = 0–3). a) Randomly oriented ceramics and b) (001)-oriented textured ceramics.


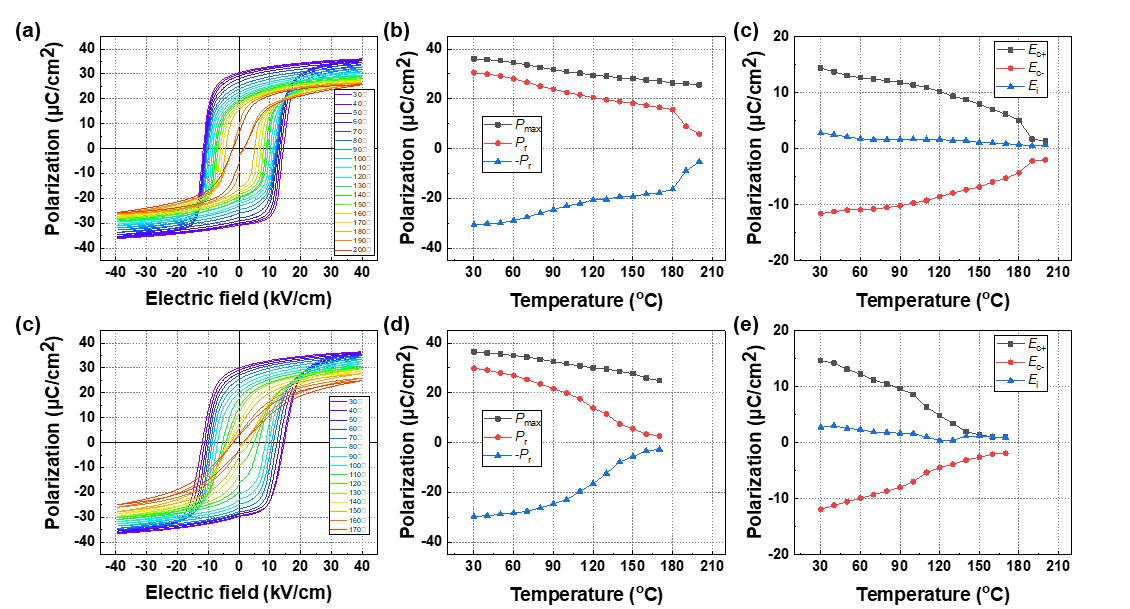


**Figure S6.** Temperature-dependent P-E curve, maximum polarization (*P*_max_), remanent polarization (*P*_r_), coercive field (*E*_c_), and internal vias (*E*_i_) of (a–c) (001)-textured Mn 2 mol% doped 0.24PIN–0.44PMN–0.32PT ceramics and (d–f) Mn 2 mol%/Eu 1.5 mol% co-doped 0.24PIN–0.46PMN–0.30PT ceramics measured at different temperatures.


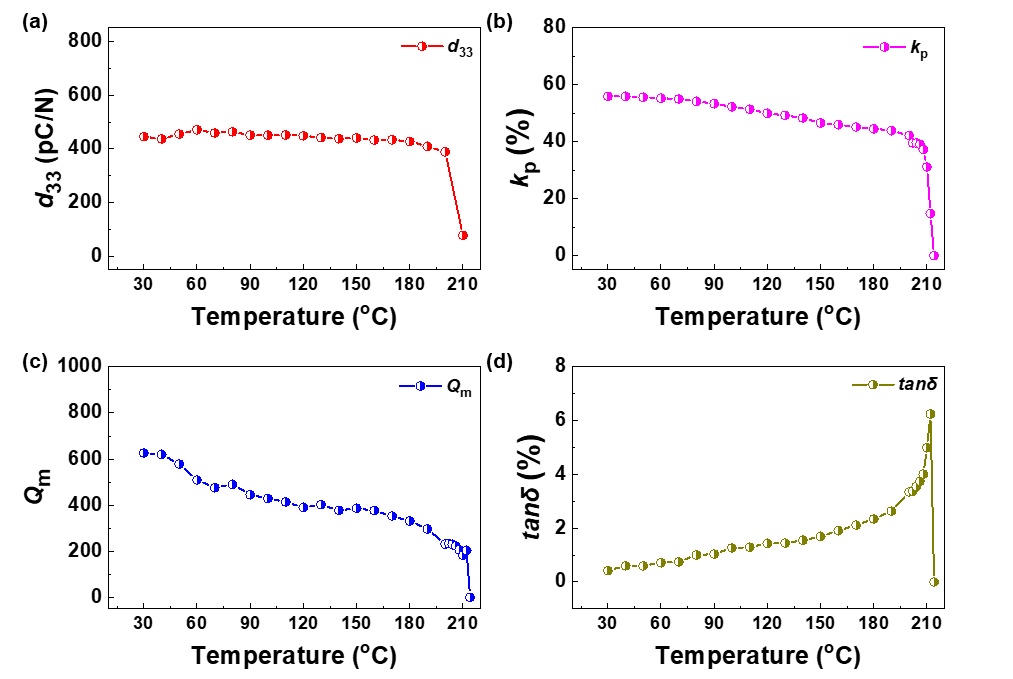


**Figure S7.** Temperature-dependent piezoelectric properties of (001)-textured Mn 2 mol% doped 0.24PIN–0.44PMN–0.32PT ceramics. The variations of (a) *d*_33_, (b) *k*_p_, (c) *Q*_m_, and (d) tan δ are shown as a function of temperature.


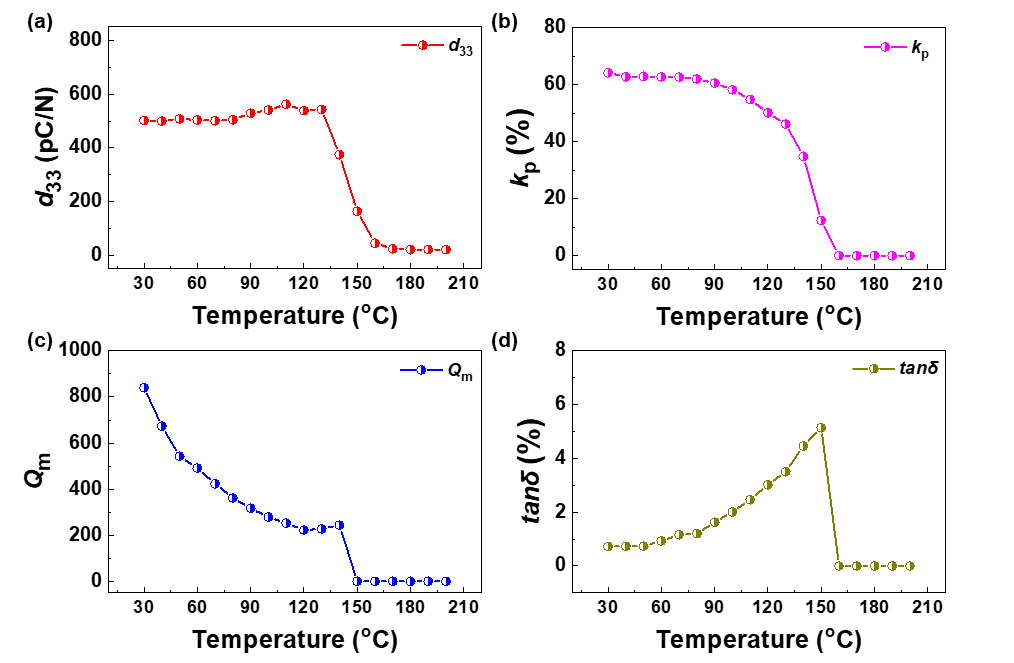


**Figure S8.** Temperature-dependent piezoelectric properties of (001)-textured Mn 2 mol%/Eu 1.5 mol% co-doped 0.24PIN–0.46PMN–0.30PT ceramics. The variations of (a) *d*_33_, (b) *k*_p_, (c) *Q*_m_, and (d) tan δ are shown as a function of temperature.

**Figure S9.** Bipolar strain–electric field (S–E) curves of poled (001)-textured Mn 2 mol% doped 0.24PIN–0.44PMN–0.32PT ceramics and Mn 2 mol%/Eu 1.5 mol% co-doped 0.24PIN–0.46PMN–0.30PT ceramics measured at room temperature.
